# Clinical and Functional Outcomes After Arthroscopic Bankart Repair After a Median Follow-up of 23 Years

**DOI:** 10.1177/03635465251388108

**Published:** 2025-11-18

**Authors:** Maximilian Hinz, Moritz Brunner, Romed P. Vieider, Kristina Zauner, Johannes E. Plath, Sebastian Lappen, Anja Wackerle, Andreas B. Imhoff, Bastian Scheiderer, Sebastian Siebenlist, Lucca Lacheta

**Affiliations:** * Department of Sports Orthopaedics, Technical University of Munich, Munich, Germany; † Orthopedic Specialist Center Weilheim/Garmisch/Starnberg/Penzberg, Weilheim, Germany; Investigation performed at the Department of Sports Orthopaedics, Technical University of Munich, Munich, Germany

**Keywords:** anterior shoulder instability, arthroscopy, shoulder surgery, revision, dislocation, instability, risk factor

## Abstract

**Background:**

Previous studies have shown that arthroscopic Bankart repair (ABR) for the treatment of anterior shoulder instability (ASI) may lead to high rates of instability recurrence and revision surgery at 10-year follow-up, but data on 20-year postoperative outcomes are scarce.

**Purpose/Hypothesis:**

The purpose was to evaluate the clinical and functional outcomes after ABR for the treatment of ASI at long-term follow-up. It was hypothesized that ABR would be associated with high rates of revision surgery, reinstability, and redislocation, but good to excellent shoulder function.

**Study Design:**

Case series; Level of evidence, 4.

**Methods:**

Patients who underwent ABR for the treatment of ASI at least 20 years ago and had initially been followed up after a minimum 10 years postoperatively were eligible to participate. Rates of revision surgery, subjective reinstability, and redislocations were evaluated, as were patient-reported outcome measures, including the American Shoulder and Elbow Surgeons score and Constant-Murley score.

**Results:**

In total, 82 patients were followed up at a median 23.0 years (IQR, 23.0-25.0) postoperatively. Twenty-eight patients (34.1%) had any kind of instability recurrence: 13 (15.9%) had reinstability at follow-up, 9 (11.0%) reported redislocations postoperatively, and 6 patients (7.3%) had both. Of those patients, 12 (42.9%) did not report instability at the previous minimum 10-year follow-up. Six patients who experienced redislocations underwent further surgery. Ten more patients underwent revision surgery for reasons other than redislocation. All patients who underwent revision surgery (n = 16; 19.5%) were excluded from further analysis. In patients who did not undergo revision surgery, shoulder function was good to excellent (median [IQR]; American Shoulder and Elbow Surgeons score, 95.0 [88.5-100]; Constant-Murley score, 87.5 [76.8-95.0]). Inferior glenohumeral laxity was associated with subjective reinstability (odds ratio, 7.214 [95% CI, 1.266-41.096]; *P* = .026). The use of fewer suture anchors for ABR was associated with redislocations (median [IQR]; no redislocation, 3.0 [3.0-4.0]; redislocation, 3.0 [2.0-3.0]; *P* = .016).

**Conclusion:**

About 1 in 3 patients reported instability recurrence or redislocations, and 1 in 5 underwent further surgery. In patients who did not undergo further surgery, good to excellent shoulder function as well as low pain and instability levels were observed at a minimum 20 years after ABR. The presence of inferior glenohumeral laxity was associated with a higher risk for subjective reinstability, and the use of fewer anchors was associated with redislocations.

Shoulder dislocations occur with an overall incidence of 0.24 to 1.69 per 1000 person-years and predominantly affect young male patients.^21,32^ As nonoperative treatment after first-time anterior shoulder dislocations leads to high rates of instability recurrence,^17,24^ surgical reattachment of the capsuloligamentous complex, namely Bankart repair,^3^ has been recommended instead.^2^ Although arthroscopic Bankart repair (ABR) is superior to nonoperative treatment, 6.7% of patients experience instability recurrence at short-term follow-up.^2^ Ten years postoperatively, the instability recurrence rate may increase to 31.2%, with 16.0% of patients experiencing recurrent dislocations despite good functional outcomes.^19^ Rossi et al^26^ recently stressed the necessity for long-term follow-up studies to avoid missing failures that occur past short-term follow-up. Several studies have reported 10-year outcomes after ABR,^1,7,8,12,14,22,25,33,35^ but there exists a paucity on outcome studies exceeding 20-year follow-up after ABR.

Therefore, a cohort that was previously assessed at a minimum 10 years after ABR for the treatment of anterior shoulder instability (ASI) was followed up again after a minimum 20 years.^1^ It was hypothesized that ABR would lead to good to excellent shoulder function and low pain levels as well as favorable sporting and working ability but that high rates of revision surgery, subjective instability, and redislocation would be observed, which have been reported at 10-year follow-up.^19^ Furthermore, established patient- and surgery-related risk factors for postoperative reinstability, including young age,^5,11,31,34^ male gender,^34^ hyperlaxity,^5,6,34^ and fewer suture anchors used,^6^ would be confirmed at minimum 20-year follow-up.

## Methods

This retrospective review study was conducted with approval from the institutional review board of the Technical University of Munich (reference 2011-51972-S-KK) and according to the Declaration of Helsinki. Patients who underwent ABR, for which the outcomes were previously reported at a minimum 10 years postoperatively,^1^ were followed up again at a minimum 20 years postoperatively. ABR was indicated in patients with symptomatic ASI. ABR was not considered in patients with large glenoid or humeral bone defects. Patients were excluded if they had concomitant rotator cuff tears, multidirectional instability, voluntary shoulder instability, and neurologic disorders involving the shoulder girdle. Data were collected via mail by a single investigator who was not involved in patient treatment.

### Surgical Technique

All surgical procedures were performed or directly supervised by the most senior surgeon (A.B.I.). A detailed description of the surgical technique has been published.^13,29^ Briefly, ABR was performed in a beach-chair position with a deep anterior-inferior working portal at the 5:30 clockface position. The absence of large glenoid defects (>25%) or engaging Hills-Sachs lesions was confirmed via diagnostic arthroscopy. Three types of hard-body anchors (n = 3.0; IQR, 3.0-4.0) were used for reconstruction during the inclusion period. Implant choice was solely based on product availability during the inclusion period. Products were used sequentially: Suretac anchors (Smith & Nephew plc) first, followed by Panalok anchors (Johnson & Johnson) and then FASTak/Bio-FASTak anchors (Arthrex Inc). The amount of capsular shift was determined via arthroscopic examination. Repair of superior labral anterior to posterior tears was performed with 2 suture anchors, if indicated. Additional rotator interval closure and capsular plication were performed with polydioxanone suture in selected cases with large capsular volume.

### Postoperative Rehabilitation

Immediately postoperatively, the operated shoulder was secured in a shoulder sling for the first 24 hours after surgery. For the first 6 postoperative weeks, abduction, flexion, and external rotation were limited to varying degrees over time. For the most part, abduction was limited to 45° from the first until the third postoperative week. This was increased to 90° between the fourth and sixth postoperative weeks. Flexion was limited to 30° and 60°, respectively, and external rotation to 60° and 75°. Patients with concomitant superior labral anterior to posterior repair were instructed not to perform resisted elbow flexion and supination during this time. From the seventh postoperative week, full range of motion was encouraged. Clearance for full return to activity was given approximately 6 months postoperatively.

### Patient Characteristics and Surgical Data

Chart review was performed to obtain patient and injury characteristics, consisting of age at the time of surgery, sex, laterality, body mass index, presence of inferior glenohumeral laxity (defined as a positive sulcus sign),^16^ primary versus recurrent instability, primary versus revision surgery, and surgical data (eg, number of anchors used for ABR).

### Outcome Measurements

Rates of revision surgery were recorded as were subjective instability and dislocation recurrence. In patients who did not undergo revision surgery, patient-reported outcome measures were evaluated, including the Constant-Murley score (CMS), the American Shoulder and Elbow Surgeons (ASES) score, and the visual analog scale (VAS) for pain and instability at rest and during activity, as well as subjective satisfaction with the postoperative result (1-10 scale, 10 = highest satisfaction). Additionally, patients were asked how surgery affected their sporting and working ability and whether they would undergo the same procedure again.

### Statistical Analysis

Data were analyzed in SPSS 28.0 (IBM). The outcomes of 1 shoulder per patient were reported and considered for statistical analyses. All patients who participated in the previous study were considered for inclusion and a power analysis was not carried out. Categorical variables are presented in number and corresponding percentage. Normal distribution of the collected continuous variables was assessed via by the Kolmogorov-Smirnov test and graphically confirmed. Normally distributed continuous variables are reported as mean and standard deviation, whereas nonnormally distributed continuous variables are shown as median and interquartile range. For group comparisons of normally distributed continuous variables, an unpaired *t* test was applied; for group comparisons of nonnormally distributed continuous or ordinal variables, the Wilcoxon signed rank test or Mann-Whitney *U* test was used. For group comparisons of categorical variables, the chi-square test was applied. Binary logistic regression analysis was performed to assess the relative contribution of follow-up length and presence of inferior glenohumeral laxity on the presence of subjective instability at follow-up. Statistical significance was set at *P* < .05.

## Results

**Figure 1.**
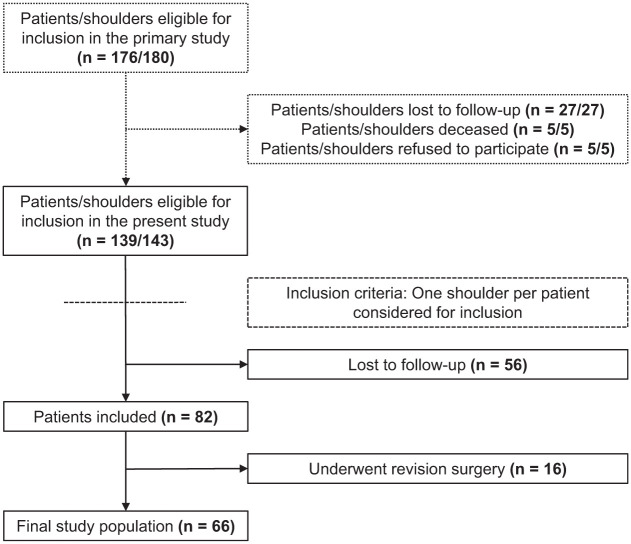
Flowchart of patient enrollment. Dotted lines represent study inclusion data from the primary study. Dashed lines represent inclusion criteria introduced in the present study.

Out of 176 originally eligible patients, 139 were included in the primary study, of which 82 were included in the present study (59.0% follow-up) at a median follow-up of 23.0 years (IQR, 23.0-25.0). Twenty-eight patients (34.1%) had any kind of instability recurrence, of which 13 (15.9%) had reinstability at follow-up, 9 (11.0%) reported redislocations postoperatively, and 6 (7.3%) reported both. Twelve of those patients (42.9%) did not have instability by the previous minimum 10-year follow-up. Six patients with postoperative dislocations underwent further surgery. Ten more patients underwent revision surgery for reasons other than redislocation, which could not be identified accurately. All patients who underwent revision surgery (n = 16, 19.5%) were excluded from further analysis. Details on patient enrollment, characteristics, and operative data are presented in Figure 1 and Table 1.

**Table 1:** Patient Characteristics and Operative Data of Total Study Cohort (n = 66)

	Mean ± SD, No. (%), or Median (IQR)
Age at the time of surgery, y	28.6 ± 8.1
Sex, male:female	51:15 (77.3 male)
Laterality, right:left	36:30 (54.5 right)
Body mass index	24.9 (22.5-27.2)
Follow-up, y	
First	12.0 (11.0-14.0)
Latest	23.0 (23.0-25.0)
Age at follow-up, y	
Minimum 10 y	41.7 ± 8.1
Minimum 20 y	52.6 ± 7.8
Inferior glenohumeral laxity*^a^*	8/51 (12.1)
Surgery after first-time dislocation	21 (31.8)
Previous shoulder surgery	10 (15.2)
No. of anchors used	3.0 (3.0-4.0)

a Data are available for 51 patients.

### Functional Outcome and Sporting/Working Ability

Shoulder function was good to excellent at the second follow-up (Table 2). When compared with the minimum 10-year follow-up, the ASES score did not change significantly (median [IQR], 94.8 [88.3-100] vs 95.0 [88.5-100]; *P* = .578), whereas the CMS decreased significantly (95.0 [91.0-98.0] vs 87.5 [76.8-95.0]; *P* < .001). Overall, low levels of pain and instability were observed at rest (VAS for pain, 0 [0-1.0]; VAS for instability, 0 [0-1.0]) and during activity (VAS for pain, 1.0 [0-2.0]; VAS for instability, 1.0 [0-3.0]). Satisfaction with the postoperative result was high (9.0 [7.8-10]). The majority of patients would undergo the same procedure again (n = 64; 97.0%). In most patients, sporting and working ability either improved or was maintained postoperatively (Table 3).

**Table 2:** Postoperative Patient-Reported Outcome Measures at Minimum 10- and 20-Year Follow-up*^a^*

	Follow-up, Median (IQR)		
Score	Minimum 10 y	Minimum 20 y	*P* Value
Constant-Murley	95.0 (91.0-98.0)	87.5 (76.8-95.0)	**< .001**
ASES	94.8 (88.3-100)	95.0 (88.5-100)	.578

a Bold *P* values indicate statistical significance. ASES, American Shoulder and Elbow Surgeons.

**Table 3:** Postoperative Sporting and Working Ability of Total Study Cohort (n = 62)*^a^*

Ability	No. (%)
Sporting	
Improved	28 (42.4)
Maintained	25 (37.9)
Declined	9 (13.6)
Working	
Improved	14 (21.2)
Maintained	47 (71.2)
Declined	1 (1.5)

a Patients who reported “not applicable” were excluded from analysis.

### Comparison Between Patients With and Without Subjective Reinstability

Independent risk factors associated with subjective reinstability included follow-up time (median [IQR]; subjective reinstability, 23.0 years [23.0-25.0]; no subjective reinstability, 23.0 years [22.0-24.0]; *P* = .021) and inferior glenohumeral laxity (no subjective reinstability, 9.8%; subjective reinstability, 40.0%; *P* = .018) (Table 4). Binary logistic regression showed that inferior glenohumeral laxity was associated with subjective reinstability (odds ratio, 7.214 [95% CI, 1.266-41.096]; *P* = .026) and follow-up time was not (*P* = .089). Shoulder function, pain, and instability levels as well as satisfaction with the postoperative result did not differ between groups.

**Table 4:** Characteristics of Patients Without vs With Subjective Reinstability*^a^*

	Subjective Reinstability, Mean ± SD, No. (%), or Median (IQR)		
	No (n = 49)	Yes (n = 17)	*P* Value
Age at surgery, y	27.6 ± 6.4	31.2 ± 11.7	.248
Sex, male:female	38:11 (77.6 male)	13:4 (76.5 male)	.927
Body mass index	25.1 (23.0-27.7)	24.3 (20.2-26.5)	.099
Follow-up, y	23.0 (23.0-25.0)	23.0 (22.0-24.0)	**.021**
Inferior glenohumeral laxity*^b^*	4/41 (9.8)	4/10 (40.0)	**.018**
Surgery after first-time dislocation	17 (34.7)	4 (23.5)	.394
Previous surgery	5 (10.2)	5 (29.4)	.067
No. of anchors used	4.0 (3.0-4.0)	3.0 (3.0-4.0)	.502
Constant-Murley score	85.0 (75.3-95.0)	92.5 (81.8-95.0)	.249
ASES score	95.0 (84.5-100)	97.0 (93.0-98.5)	.900
VAS			
For pain at rest	00 (0-1.0)	00 (0-1.0)	.821
For pain during activity	1.0 (0-2.0)	1.0 (0-2.0)	.894
For instability at rest	00 (0-1.0)	00 (0-2.5)	.581
For instability during activity	1.0 (0-2.5)	2.0 (0.3-5.5)	.125
Satisfaction	9.0 (8.0-10)	8.0 (1.5-10)	.075

a Bold *P* values indicate statistical significance. ASES, American Shoulder and Elbow Surgeons; VAS, visual analog scale.

b Data are available for 51 patients.

### Comparison Between Patients With and Without Redislocations

The number of anchors used for Bankart repair (median [IQR]; no redislocations, 3.0 [3.0-4.0]; redislocations, 3.0 [2.0-3.0]; *P* = .016) was the only independent risk factor associated with postoperative redislocations (Table 5). Patients with postoperative redislocations reported higher pain at rest (no redislocations, 0 [0-1.0]; redislocations, 1.0 [1.0-3.0]; *P* = .009) and higher instability levels at rest (no redislocations, 0 [0-1.0]; redislocations, 1.0 [0-4.8]; *P* = .046) and during activity (no redislocations, 1.0 [0-2.0]; redislocations, 6.0 [3.0-7.5]; *P* < .001) as well as lower satisfaction with the postoperative result (no redislocations, 9.0 [8.0-10]; redislocations, 5.0 [2.5-8.5]; *P* = .003). Shoulder function did not differ between the groups.

**Table 5:** Characteristics of Patients Without vs With Redislocations*^a^*

	Redislocations, Mean ± SD, No. (%), or Median (IQR)		
	No (n = 57)	Yes (n = 9)	*P* Value
Age at surgery, y	29.1 ± 8.1	25.3 ± 7.9	.248
Sex, male:female	45:12 (78.9 male)	6:3 (66.7 male)	.414
Body mass index	24.9 (22.5-27.3)	25.1 (21.0-26.9)	.814
Follow-up, y	23.0 (23.0-25.0)	23.0 (22.0-24.5)	.453
Inferior glenohumeral laxity*^b^*	7/45 (15.6)	1/6 (16.7)	.944
Surgery after first-time dislocation	16 (28.1)	5 (55.6)	.100
Previous surgery	9 (15.8)	1 (11.1)	.716
No. of anchors used	3.0 (3.0-4.0)	3.0 (2.0-3.0)	**.016**
Constant-Murley score	89.0 (77.5-95.0)	84.0 (65.5-92.5)	.331
ASES score	95.0 (90.0-100)	95.5 (71.8-99.5)	.548
VAS			
For pain at rest	00 (0-1.0)	1.0 (1.0-3.0)	**.009**
For pain during activity	1.0 (0-2.0)	2.0 (1.3-2.8)	.111
For instability at rest	00 (0-1.0)	1.0 (0-4.8)	**.046**
For instability during activity	1.0 (0-2.0)	6.0 (3.0-7.5)	**<.001**
Satisfaction	9.0 (8.0-10)	5.0 (2.5-8.5)	**.003**

a Bold *P* values indicate statistical significance. ASES, American Shoulder and Elbow Surgeons; VAS, visual analog scale.

b Data are available for 51 patients.

## Discussion

The most important finding of this study was that ABR for the treatment of ASI was associated with good to excellent shoulder function and improved or maintained sporting and working ability at minimum 20-year follow-up in patients who did not undergo revision surgery. High rates of revision surgery, instability, and dislocation recurrence, however, were observed. Furthermore, the presence of inferior glenohumeral laxity has been associated with higher odds for postoperative reinstability, and the use of fewer suture anchors was found in patients with postoperative redislocations. We believe that this may be due to the postoperative increase in capsular volume,^23^ which may have occurred to a greater extent in patients with inferior glenohumeral laxity than in those without, potentially leading to reinstability without redislocation more frequently in those patients. Yet, the use of fewer suture anchors may have led to a biomechanically less favorable repair and therefore a higher redislocation rate. Young age and male gender were not associated with a higher risk for reinstability or redislocation.

A significant deterioration of shoulder function via CMS was observed from the minimum follow-up of 10 years until the minimum of 20 years. Still, at both times, the median CMS was higher than the mean values of a healthy population.^9^ It may thus be assumed that this deterioration was linked to the natural aging process rather than a diminishing effect of the ABR over time.

As there exists a paucity of data on 20-year outcomes after ABR, the results of the present study were compared with data from a recent systematic review reporting on the 10-year outcomes of ABR.^19^ Murphy et al,^19^ who included 822 shoulders across 9 studies,^1,7,8,12,14,22,25,33,35^ reported high satisfaction rates (85.6%) but slightly less favorable shoulder function (weighted mean CMS, 76.2) as compared with the present study. Rates of revision (17.0%), reinstability (31.2%), and redislocations (16.0%) were comparable to the present study despite the difference in length of follow-up. These similarities may be due to most shoulder dislocations after ABR occurring at midterm follow-up.^26,35^ It should, however, be mentioned that the patients in the present study were included in the systematic review by Murphy et al, which may have influenced the presence of similarities between studies. Berendes et al^4^ and Fabre et al^10^ evaluated the outcomes of patients who underwent open Bankart repair at a mean 21 and 28 years postoperatively. Similar shoulder function and redislocation rates as compared with the present study were reported. Considerably lower reinstability and redislocation rates were noted for the Latarjet procedure at a mean follow-up of 20 years.^18^ When compared with outcomes at a median 17 years after nonoperative treatment, the instability recurrence rates were considerably lower in the present study.^20^

In the present study, preoperative inferior glenohumeral laxity was identified as a risk factor for instability recurrence, and fewer suture anchors used for ABR was associated with postoperative redislocations. Laxity and the use of fewer anchors have been associated with postoperative instability recurrence in previous studies.^6,34^ Beyond these factors, several other risk factors have been reported by previous studies, such as young age,^11,31,34^ male gender,^34^ off-track Hill-Sachs lesions,^6,11,15,27,31,34^ glenoid bone loss,^6,11,31,34^ and the number of preoperative dislocations.^15^

Some limitations should be noted when interpreting the results of the present study. First, magnetic resonance imaging was not available, which limited the assessment of several confounding factors associated with subjective reinstability and redislocation, such as off-track Hill-Sachs lesions^5,6,11,15,27,31,34^ and glenoid bone loss.^6,11,31,34^ As a result, these findings—particularly glenoid bone loss—could not be precisely reported. Second, factors such as the number of preoperative dislocations,^5,15^ which may also affect the outcome, could not be analyzed owing to potential recall bias. Third, the onset or progression of instability arthropathy, previously reported as high as 59.4% at 10 years after ABR,^19^ could not be assessed, as no imaging was available at the latest follow-up and the type of revision procedures that patients underwent could not be retrieved given the long follow-up time frame. Fourth, suture anchor systems, indications for ABR,^28^ surgical techniques, and concomitant procedures, particularly remplissage,^30^ have developed since the surgical treatment of patients in this study; as such, the outcomes of patients who currently undergo ABR may differ to those of patients who underwent ABR >20 years ago. Fifth, while patient-reported outcome measures evaluated in the present study, specifically ASES score and CMS, allowed for an assessment of general shoulder function, they may not have been specific enough for the evaluation of a cohort with shoulder instability. Also, for those patients with previous surgery, the nature of the surgery was not known. Furthermore, the high loss to follow-up may have introduced bias into the results. Last, as this study was retrospective in nature, the presence of inferior glenohumeral laxity was retrieved via chart review and was not available for all patients.

Previous studies have shown that short- and midterm follow-up may not be sufficient to record dislocation recurrence after ABR,^26,35^ yet studies are scarce showing that first redislocations after ABR may occur >20 years after surgery. Patients with ASI should be informed preoperatively regarding their expected high postoperative shoulder function but also high risk for revision, reinstability, and redislocation at long-term follow-up.

## Conclusion

About 1 in 3 patients reported instability recurrence or redislocations and 1 in 5 patients underwent further surgery. In patients who did not undergo further surgery, good to excellent shoulder function as well as low pain and instability levels were observed at a minimum 20 years after ABR. The presence of inferior glenohumeral laxity was associated with a higher risk for postoperative reinstability, and the use of fewer anchors was associated with postoperative redislocations.
